# SURVIVAL TREND IN COLORECTAL CANCER IN AFRICAN AMERICAN PATIENTS OVER THE LAST DECADES

**DOI:** 10.51894/001c.122888

**Published:** 2024-08-30

**Authors:** Vihangi Niyangoda, Tarik Wasfie, Hemal Dholakia, Erica Roach, Harold Freeman

**Affiliations:** 1 College of Osteopathic Medicine Michigan State University

## 33

### BACKGROUND/OBJECTIVES

African American (AA) patients with colorectal cancer had a lower survival rate than White Caucasian patients regardless of the stage. We aim in this study to compare our data of colorectal cancer survival of AA patients during 1963-1986 and compare it to recently published data on a similar group of patients.

### METHODS

This is a retrospective analysis of patients with colorectal cancer. Data collected and analyzed were age, sex, race, insurance carriers, stage of the cancer at presentation to treatment, treatment provided, and survival rate at 5 and 10 years. These results are compared with recent available and published data with similar groups of African American patients with colorectal cancer.

### RESULTS

There was a total of 803 patients. Of those, 754 (94%) patients were AA with the mean age of the group 68 years. Females made-up 56% and males made-up 44%. Of the 635 patients the five-year survival rate was 19%. Compared to published data, the five-year survival rate from colon cancer increased slightly among AA diagnosed in early 2000 but has not reached the level of that of WC (male and female AA= 22.7 and 14.8/100,000 vs male and female WC=15.8 and 11.3/100,000, p<0.001).

### CONCLUSION

These findings suggest that disparities in survival between AA patients with colorectal cancer remain high compared to White Caucasians, despite the efforts to close the differences by increased screening and improved accessibility to healthcare services. Therefore, we should maximize the existing effort and develop different strategies to reduce the existing differences.

